# Supplementary material for: Enhancing carbohydrate repartitioning into lipid and carotenoid by disruption of microalgae starch debranching enzyme
Source: Commun Biol. 2021 Apr 9;4:450. doi: 10.1038/s42003-021-01976-8 (PMC8035404; doi:10.1038/s42003-021-01976-8)
Supplement: Supplementary file 2 — Supplementary Information [file 42003_2021_1976_MOESM2_ESM.pdf]

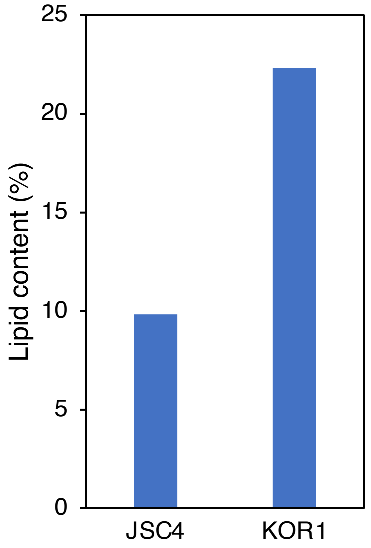

Supplementary Figure 1. Lipid content of KOR1 in the secondary screening. Candidate mutant strains were cultured in 12 well plates under illumination of white LEDs (100 µmol photons/m^2/s, 12 h:12 h light/dark cycling), 2 % CO_2, 30 ° C, 100 rpm, at an initial cell concentration of OD_{750} = 0.1. The mutant strains were grown with MB 6N containing 2 % (*w/v*) sea salt for initial 4 days, and then resuspended in MB 0N (MB medium without $NaNO_3$) containing 2 % (*w/v*) sea salt and further cultured for 5 days. Cultured cells were harvested, lyophilized, and subjected to lipid measurement using GC-MS.

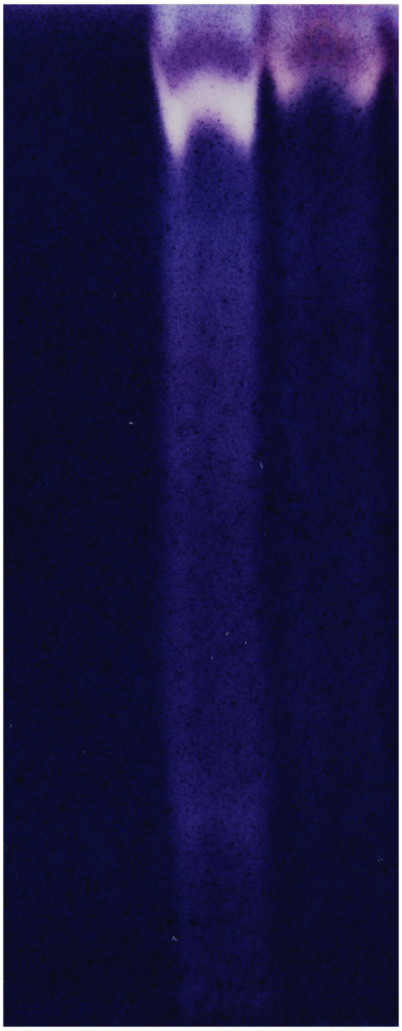

Supplementary Figure 2. Zymography for DBE activity. Cells at day 4.0 were suspended in extraction buffer (50 mM imidazole-HCl (pH = 7.4), 8 mM $MgCl_2$, 50 mM 2-mercaptoethanol, and 12.5 % (*v/v*) glycerol), frozen and thawed with liquid nitrogen and 30 ° C water four times, and then sonicated (output 4, duty 50 %, 5 min). The suspension was centrifuged at 20,000 × *g* for 20 min at 4 ° C. Native-PAGE was performed using the supernatant containing 10 μg of protein and an acrylamide gel containing 0.8 % (*w/v*) potato amylopectin with a constant current of 15 mA. After electrophoresis, the gel was incubated in reaction buffer (50 mM citric-Na_2HPO_4 (pH = 6.0) and 50 mM 2-mercaptoethanol) at 30° C for 2 h. DBE activity was detected by staining the gel with 0.1% (*w/v*) I_2 / 1% (*w/v*) KI solution.

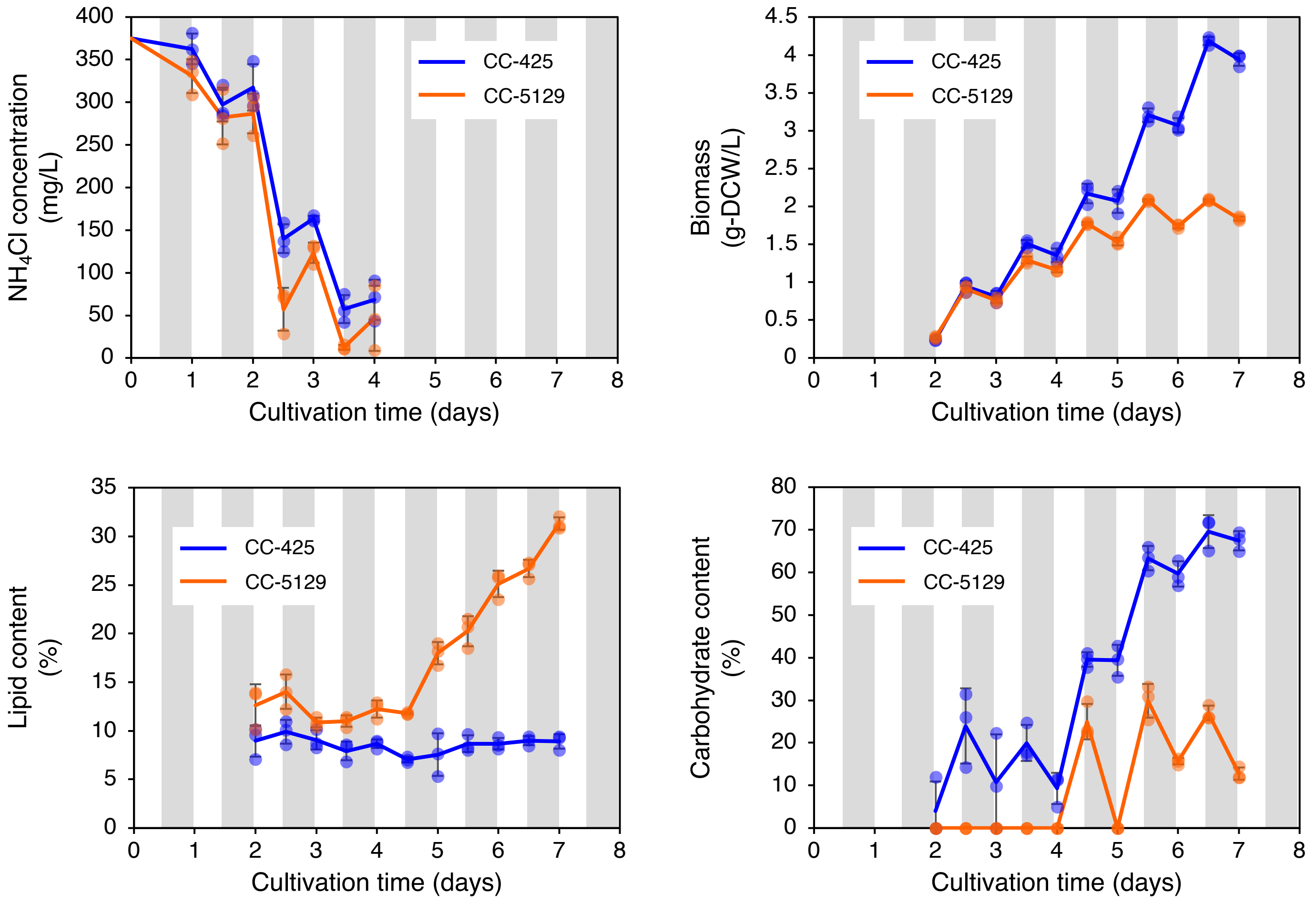

Supplementary Figure 3. Time-course profiles of NH_4Cl concentration, biomass, lipid content, and carbohydrate content of *Chlamydomonas reinhardtii sta7-10* mutant (CC-5129) and its parent strain (CC-425). Microalgae were cultured in TAP medium using double-deck photobioreactors under illumination of white fluorescent lamps (250 µmol photons/m^2/s, 12 h:12 h light/dark cycling), 2 % CO_2, 30 ° C, 100 rpm, at an initial cell concentration of 20 mg/g-DCW. White and gray bands represent light and dark periods, respectively. Error bars indicate the standard deviation of three replicate experiments

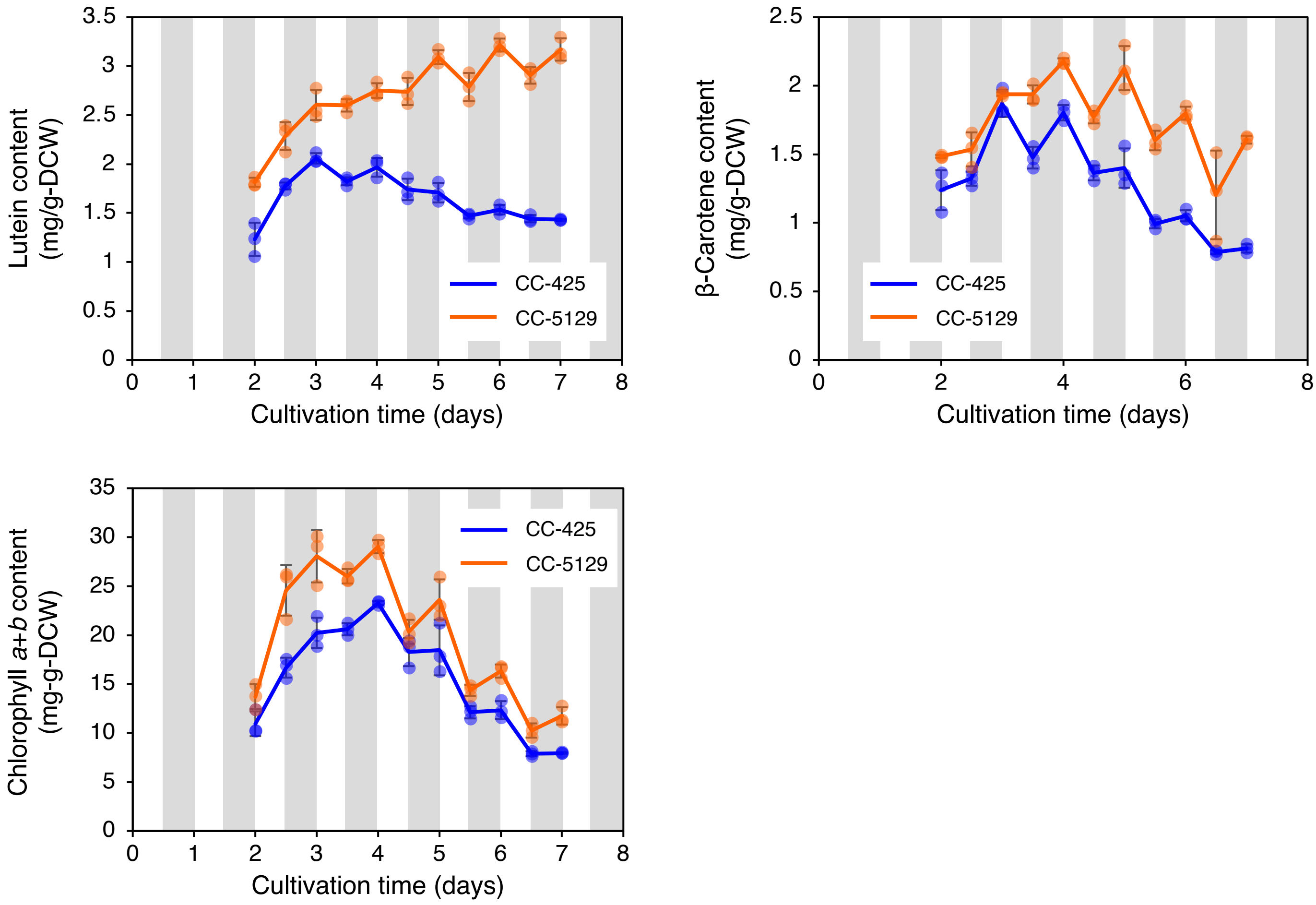

Supplementary Figure 4. Time-course profiles of lutein content, β-carotene content, and chlorophyll *a+b* content of *Chlamydomonas reinhardtii sta7-10* mutant (CC-5129) and its parent strain (CC-425). Microalgae were cultured in TAP medium using double-deck photobioreactors under illumination of white fluorescent lamps (250 µmol photons/m^2/s, 12 h:12 h light/dark cycling), 2 % CO_2, 30 ° C, 100 rpm, at an initial cell concentration of 20 mg/g-DCW. White and gray bands represent light and dark periods, respectively. Error bars indicate the standard deviation of three replicate experiments.

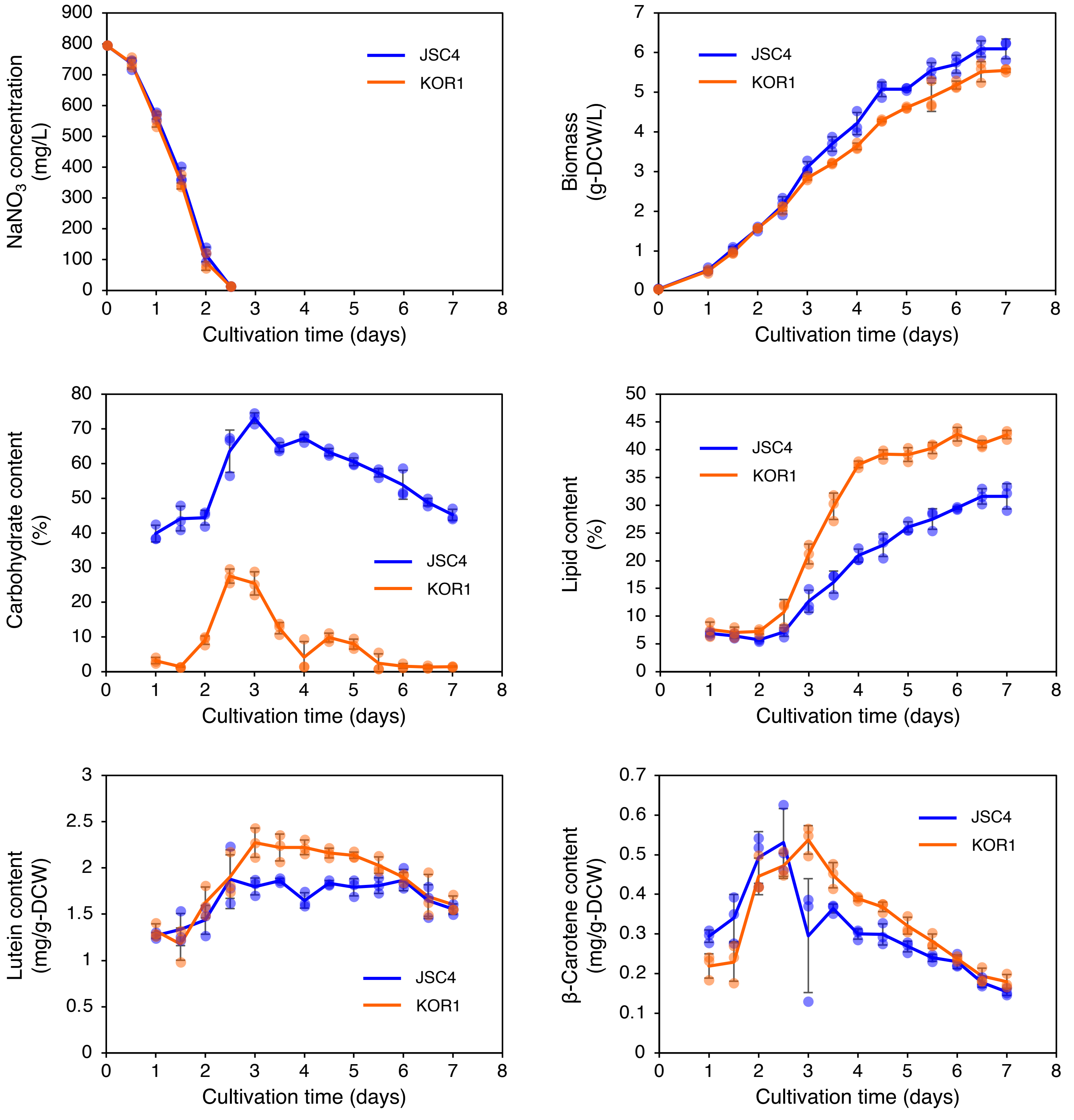

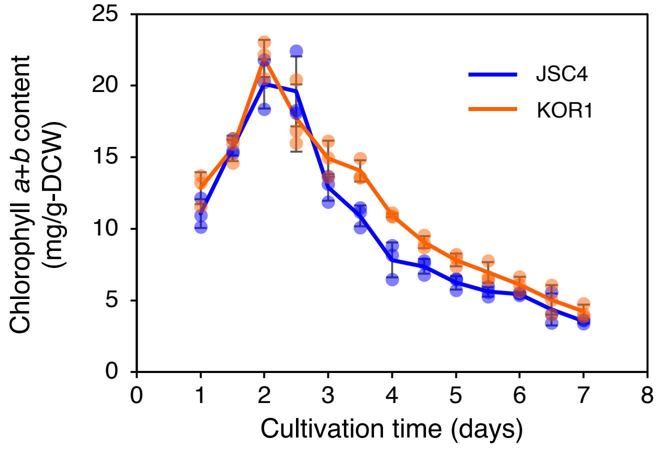

Supplementary Figure 5. Time-course profiles of NaNO$_3$ concentration, biomass, carbohydrate content, lipid content, lutein content, β-carotene content, and chlorophyll *a+b* content under continuous illumination. Microalgae were cultured in MB 6N containing 2 % (*w/v*) sea salt medium using double-deck photobioreactors under illumination of white fluorescent lamps (250 μmol photons/m^2/s, 12 h:12 h light/dark cycling), 2 % CO_2, 30 ° C, 100 rpm, at an initial cell concentration of 20 mg/g-DCW. Error bars indicate the standard deviation of three replicate experiments.

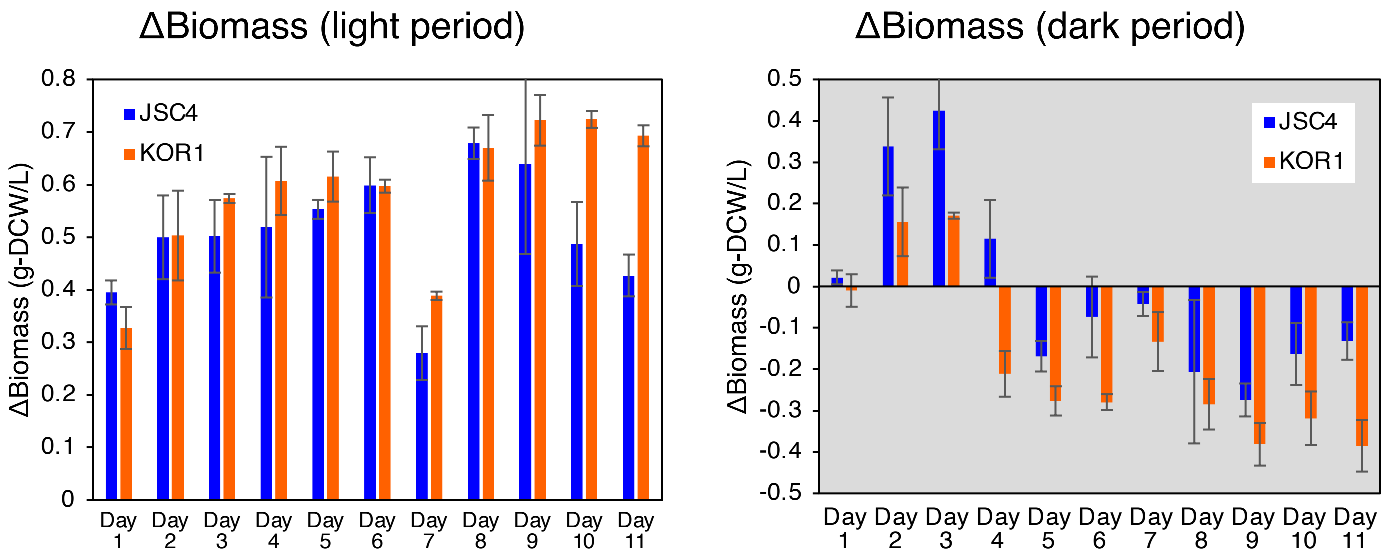

Supplementary Figure 6. Changes in biomass throughout light periods and dark periods. Microalgae were cultured in MB 6N containing 2 % (*w/v*) sea salt medium using double-deck photobioreactors under illumination of white fluorescent lamps (250 μmol photons/m^2/s, 12 h:12 h light/dark cycling), 2 % CO_2, 30 ° C, 100 rpm, at an initial cell concentration of 20 mg/g-DCW. Error bars indicate the standard deviation of three replicate experiments.

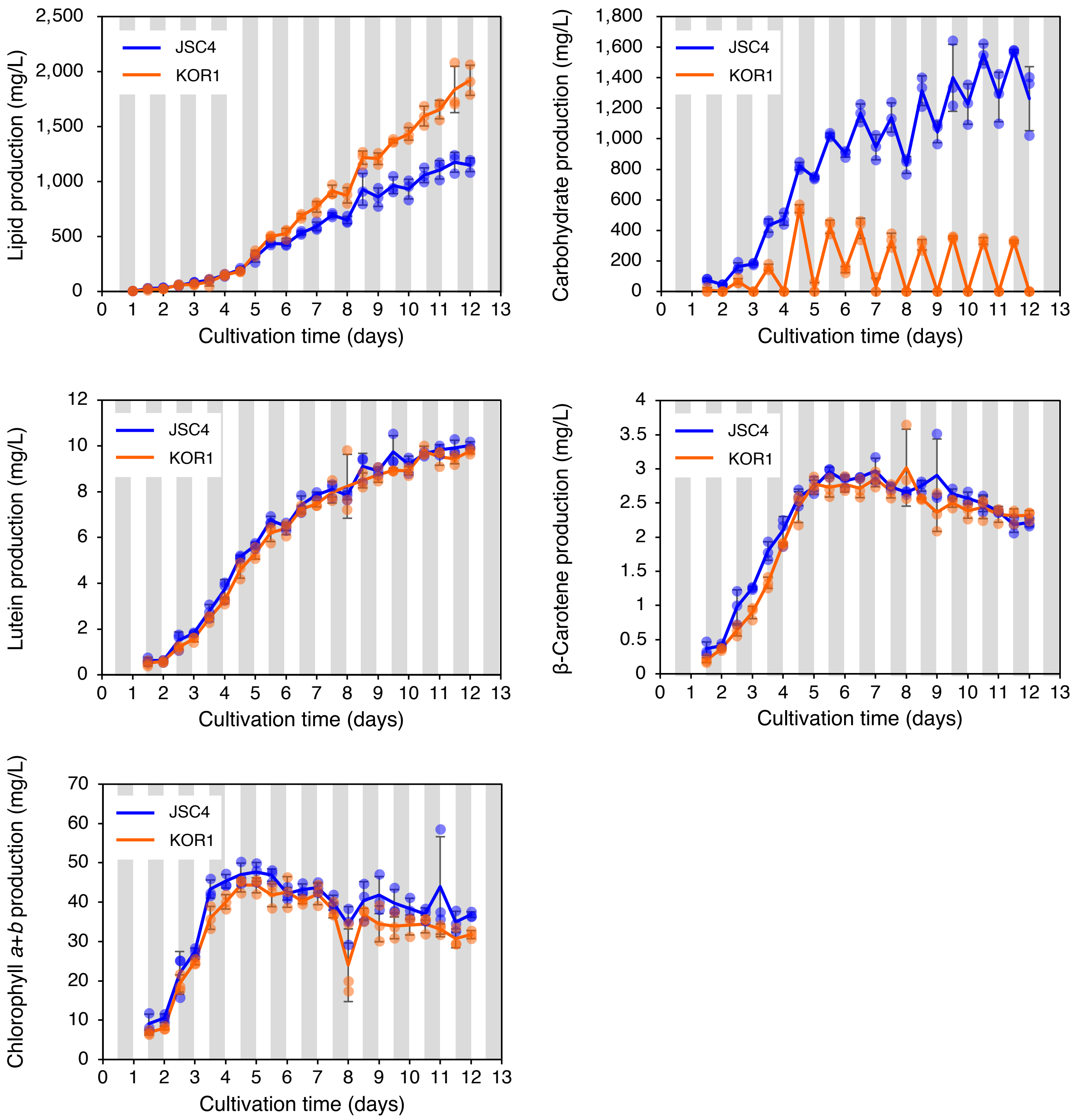

Supplementary Figure 7. Time-course profiles for culture volume-based production of lipid, carbohydrate, lutein, β-carotene, and chlorophyll *a+b*. Microalgae were cultured in MB 6N containing 2 % (*w/v*) sea salt medium using double-deck photobioreactors under illumination of white fluorescent lamps (250 µmol photons/m^2/s, 12 h:12 h light/dark cycling), 2 % CO_2, 30 ° C, 100 rpm, at an initial cell concentration of 20 mg/g-DCW. White and gray bands represent light and dark periods, respectively. Error bars indicate the standard deviation of three replicate experiments.
