# Supplementary material for: Enhancing carbohydrate repartitioning into lipid and carotenoid by disruption of microalgae starch debranching enzyme
Source: Commun Biol. 2021 Apr 9;4:450. doi: 10.1038/s42003-021-01976-8 (PMC8035404; doi:10.1038/s42003-021-01976-8)
Supplement: Supplementary file 3 — Description of Additional Supplementary Files [file 42003_2021_1976_MOESM3_ESM.pdf]

Description of Additional Supplementary Files

File Name: Supplementary Data 1

Description: The source data underlying Fig. 1, Fig. 4, Fig. 5, and Fig. 6.
